# Rilonacept in the treatment of acute gouty arthritis: a randomized, controlled clinical trial using indomethacin as the active comparator

**DOI:** 10.1186/ar4159

**Published:** 2013-02-01

**Authors:** Robert A Terkeltaub, H Ralph Schumacher, John D Carter, Herbert SB Baraf, Robert R Evans, Jian Wang, Shirletta King-Davis, Steven P Weinstein

**Affiliations:** 1 Division of Rheumatology, Allergy and Immunology, VA Healthcare System San Diego and University of California at San Diego School of Medicine, 3350 La Jolla Village Drive, San Diego, CA 92161, USA; 2 VA Medical Center and University of Pennsylvania, 151K, University & Woodland Avenues, Philadelphia, PA 19104, USA; 3 Division of Rheumatology, University of South Florida, 12901 Bruce B Downs Blvd, Tampa, FL 33612, USA; 4 The Center for Rheumatology and Bone Research, 2730 University Boulevard West, Suite 310, Wheaton, MD 20902, USA; 5 Clinical Sciences, Regeneron Pharmaceuticals, Inc., 777 Old Saw Mill River Road, Tarrytown, NY 10591, USA; 6 BioStatistics, Regeneron Pharmaceuticals, Inc., 777 Old Saw Mill River Road, Tarrytown, NY 10591, USA; 7 Clinical Trial Management, Regeneron Pharmaceuticals, Inc., 777 Old Saw Mill River Road, Tarrytown, NY 10591, USA

## Abstract

**Introduction:**

In phase-3 clinical trials, the interleukin (IL-1) blocker, rilonacept (IL-1 Trap), demonstrated efficacy for gout flare prevention during initiation of urate-lowering therapy. This trial evaluated rilonacept added to a standard-of-care, indomethacin, for treatment of acute gout flares.

**Methods:**

Adults, aged 18-70 years, with gout presenting within 48 hours of flare onset and having at least moderate pain as well as swelling and tenderness in the index joint were randomized to subcutaneous (SC) rilonacept 320 mg at baseline plus oral indomethacin 50 mg TID for 3 days followed by 25 mg TID for up to 9 days (*n *= 74); SC placebo at baseline plus oral indomethacin as above (*n *= 76); or SC rilonacept 320 mg at baseline plus oral placebo (*n *= 75). The primary efficacy endpoint was change in pain in the index joint (patient-reported using a Likert scale (0 = none; 4 = extreme)) from baseline to the average of values at 24, 48 and 72 hours (composite time point) for rilonacept plus indomethacin versus indomethacin alone. Comparison of rilonacept monotherapy with indomethacin monotherapy was dependent on demonstration of significance for the primary endpoint. Safety evaluation included clinical laboratory and adverse event (AE) assessments.

**Results:**

Patient characteristics were comparable among the groups; the population was predominantly male (94.1%), white (75.7%), with mean ± SD age of 50.3 ± 10.6 years. All treatment groups reported within-group pain reductions from baseline (*P *< 0.0001). Although primary endpoint pain reduction was greater with rilonacept plus indomethacin (-1.55 ± 0.92) relative to indomethacin alone (-1.40 ± 0.96), the difference was not statistically significant (*P *= 0.33), so formal comparison between monotherapy groups was not performed. Pain reduction over the 72-hour period with rilonacept alone (-0.69 ± 0.97) was less than that in the other groups, but pain reduction was similar among groups at 72 hours. Treatment with rilonacept was well-tolerated with no reported serious AEs related to rilonacept. Across all groups, the most frequent AEs were headache and dizziness.

**Conclusions:**

Although generally well-tolerated, rilonacept in combination with indomethacin and rilonacept alone did not provide additional pain relief over 72 hours relative to indomethacin alone in patients with acute gout flare.

**Trial registration:**

ClinicalTrials.gov registration number NCT00855920.

## Introduction

A cardinal clinical feature of gout is recurrent acute inflammatory flares (acute gout flares) that result in debilitating joint pain and swelling. Gouty arthritis is mediated by monosodium urate monohydrate crystal deposition in and around the joint space due to hyperuricemia. Acute gout flares can be precipitated by a variety of factors including joint trauma, and putative remodeling of articular crystal deposits due to changes in serum urate concentrations, such as during the early months of initiation of uric acid-lowering therapy (ULT) [1,2].

The incidence and prevalence of gout are increasing, partly as a consequence of increased prevalence of comorbidities such as metabolic syndrome, type II diabetes, obesity, hypertension, and chronic kidney disease [3,4]. Gout is associated with a substantial economic burden due to high health care resource utilization and reduced work productivity [5-7], especially among patients who are refractory to conventional gout management strategies [8,9].

Because of their anti-inflammatory and analgesic characteristics, non-steroidal anti-inflammatory drugs (NSAIDs) are often used as first-line therapy for the treatment of acute gout flares [10,11]. Colchicine and systemic and locally injected corticosteroids are also appropriate options in many patients [10-12], with the corticosteroid prednisolone in particular showing equivalent efficacy to the NSAID naproxen [13]. However, colchicine is associated with risks of toxicity especially related to renal impairment and drug-drug interactions [14], and NSAIDs are also associated with clinically recognized risks of toxicities, especially related to their gastrointestinal and cardiovascular effects [15,16]. In a recent study, more than 90% of gout patients had a relative or absolute contraindication to NSAIDs, and up to 66% of patients had a contraindication to colchicine or a condition warranting colchicine dose reduction [17]. The presence of comorbid conditions in these patients included hypertension (88.7%), coronary artery disease (37.4%), chronic kidney disease (47.1%), and gastroesophageal disease (> 20%), with 65% of patients having multiple comorbidities [17]. Such risks increase among individuals with comorbidities and in those taking multiple medications, circumstances that are common in older adults [18,19]. Moreover, the intense pain of gout attacks is reduced with NSAID, colchicine, or corticosteroid therapy by only approximately 50% in 1 to 3 days in most clinical trials [12,13,20,21]. Consequently, there is a need for new approaches that provide increased efficacy and/or tolerability in the treatment of acute gouty arthritis.

IL-1β is a major mediator of gouty inflammation and pain [22], and is now being increasingly evaluated for its role in acute and chronic gout. Of particular relevance is the observation that monosodium urate (MSU) crystals induce activation of the NLRP3 inflammasome, a protein complex expressed in macrophages and certain other cell types, which promotes caspase-1-driven release of mature IL-1β, with subsequent induction of numerous downstream inflammatory mediators that contribute to the clinical presentation of the signs and symptoms of gouty arthritis [23]. Neutrophils and mast cells also express proteases such as elastase and chymase, respectively, that activate pro-IL-1β [24]. Data from case reports and early-phase clinical trials of the IL-1 inhibitors anakinra and canakinumab confirmed the role of IL-1 inhibition as a treatment option for acute gout [25-30]. In particular, studies of the IL-1β-specific monoclonal antibody canakinumab for the treatment of an acute gout flare provided evidence of efficacy relative to a single intramuscular (IM) low dose of triamcinolone acetonide 40 mg [30,31].

Rilonacept is a fully-human, recombinant, soluble decoy receptor protein engineered from human IL-1 receptors and IgG1Fc that binds IL-1α and IL-1β, thus preventing their activation of cell surface receptors [32]. Since rilonacept was generated using so-called Trap Technology, it is also known as the IL-1 Trap [32]. The half-life of rilonacept is approximately 1 week [33]. Recent clinical trials of rilonacept in gout have demonstrated significant and marked efficacy vs placebo for prevention of acute gout flare over 4 months, among patients initiating ULT with allopurinol [34,35]. We therefore tested in the current study the hypothesis that a single subcutaneous (SC) administration of rilonacept, along with an oral NSAID, during the first 48 hours of an acute gout flare can reduce the pain of the gout attack compared to NSAID alone. Specifically, in this superiority study, we evaluated the efficacy and safety of SC rilonacept as add-on therapy to a standard-of-care NSAID regimen of oral indomethacin, rilonacept monotherapy, and indomethacin monotherapy, for the reduction of pain in patients experiencing an acute gout flare.

## Materials and methods

### Study design and patient population

This phase 3, randomized, double blind, double-dummy, active- and placebo-controlled study was conducted at 60 study centers in North America. The study was performed in accordance with the revised Declaration of Helsinki, approval was obtained from the independent ethics committee (Copernicus Group IRB, One Triangle Drive, Suite 100, Research Triangle Park NC 27709), and all patients provided written informed consent.

The study was open to male and female patients 18 to 70 years of age having a diagnosis of primary gout based on the American Rheumatism Association (1977 ARA preliminary criteria) for the classification of acute arthritis of primary gout [36]. Eligible patients must also have previously demonstrated symptomatic relief with NSAIDs for treatment of gout flare. Exclusion criteria included, but were not limited to treatment with short-acting NSAIDs within 48 hours of randomization, or other NSAIDs based on duration of action; use of systemic glucocorticoids within 4 weeks of randomization; use of colchicine at a dose exceeding 0.6 mg twice daily within 7 days of randomization; a history of NSAID intolerance or absolute contraindication; active or recurrent infections, and estimated creatinine clearance < 60 mL/minute using the Cockcroft-Gault method [37]. Since NSAID use was required in this study, a history of bleeding disorders, gastrointestinal bleeding or perforation, as well as poorly controlled hypertension and other cardiovascular risk factors were reasons for exclusion.

Eligible patients remained in screening and were subsequently randomized to treatment when they presented with an acute flare within 48 hours of pain onset and met the following additional criteria: pain in the gouty index joint of at least moderate severity using a 5-point Likert scale (1 = none, 2 = mild, 3 = moderate, 4 = severe, 5 = extreme), a score of at least 1 on a scale of 0 to 3 for assessments of swelling and tenderness at the gouty index joint, and presentation of acute gout flare in three joints or fewer. The index joint was defined as the joint that was most painful at the time of randomization.

### Randomization and dosing

Patients were randomly allocated 1:1:1 to treatment with either SC placebo at baseline plus oral indomethacin 50 mg three times daily for 3 days (and then 25 mg three times daily for up to 9 days); SC rilonacept 320 mg at baseline plus oral indomethacin as above; or SC rilonacept 320 mg at baseline plus oral placebo three times daily for 3 days and then oral placebo three times daily for up to 9 days. Randomization was stratified by baseline pain score (moderate, or severe or greater) and country. On-site study visits occurred at baseline (day 1), days 4 and 8, and at a safety follow up on day 31.

### Endpoints

Patient self-assessment of pain and other gout symptoms were recorded using an electronic diary at baseline, 4, 8, 12, and 24 hours, and then daily until the flare ended. Pain was assessed using the 5-point Likert scale and an 11-point numerical rating scale (NRS; 0 = no pain to 10 = extreme pain).

Based on index joint pain, patients were eligible for rescue medication at two time points. At 24 hours, patients with extreme pain or severe pain that had not decreased from baseline were eligible for rescue medication with blinded indomethacin 50 mg three times daily for 1 day (group not receiving indomethacin, that is, rilonacept only group) or blinded placebo rescue in the other two treatment groups receiving indomethacin. At 48 hours patients with either a) extreme pain alone or b) moderate or severe pain and pain reduction from baseline less than 20% were eligible to receive blinded rescue with indomethacin in the rilonacept-only group (50 mg three times daily for 1 day, followed by 25 mg three times daily for up to 9 days), or with placebo in the two groups receiving indomethacin; the duration of rescue was determined by the investigator. Patients receiving rescue medication were not required to be withdrawn. For all groups, efficacy data were set to missing after initiation of rescue medication. The last observation carried forward (LOCF) method was used to impute data after rescue medication for the analysis of covariance (ANCOVA) model, used for the primary analysis.

The primary efficacy endpoint was the change in patient-reported pain (Likert scale) in the index joint from baseline to the average of the patient-reported pain values at 24 (day 2), 48 (day 3) and 72 hours (day 4) (composite time point). Secondary efficacy endpoints included the change from baseline in patient-reported pain in the index joint at days 2, 3, and 4. Additional exploratory analyses included proportion of patients requiring rescue medication. Blood samples were obtained for analysis of high sensitivity C-reactive protein (hs-CRP), an established marker of inflammation, at baseline and at study visits on days 4, 8, and 31. Safety and tolerability were evaluated based on the incidence of adverse events (AEs) up to and including the safety follow up visit, and the occurrence of clinically significant laboratory findings determined by the investigator.

### Statistical analysis

For this superiority study, a pre-specified step-down sequential testing procedure was used for the primary efficacy endpoint that compared rilonacept alone vs indomethacin alone only if the comparison of rilonacept + indomethacin vs indomethacin alone was statistically significant (*P *< 0.05). ANCOVA was used for the sequential testing of endpoints with treatment and baseline pain score strata (moderate, or severe or greater) and country as fixed effects. The LOCF was used for imputation of missing data, and pain scores obtained after rescue medication were considered missing for the primary efficacy analysis.

A sample size of 75 patients per group was calculated to provide at least 90% power for pairwise comparisons based on the following assumptions: a 1-point improvement from baseline for the control group; a 2-point change in the comparator group, and a constant SD of 0.85, for 2-sided alpha level of 0.05 for the comparisons.

All analyses were performed using SAS Version 9 (SAS Institute, Cary, NC, USA). The full analysis set included all randomized patients who received any study medication and had at least one post-baseline assessment, and the safety set included all patients who received any study medication.

## Results

**Figure 1:**
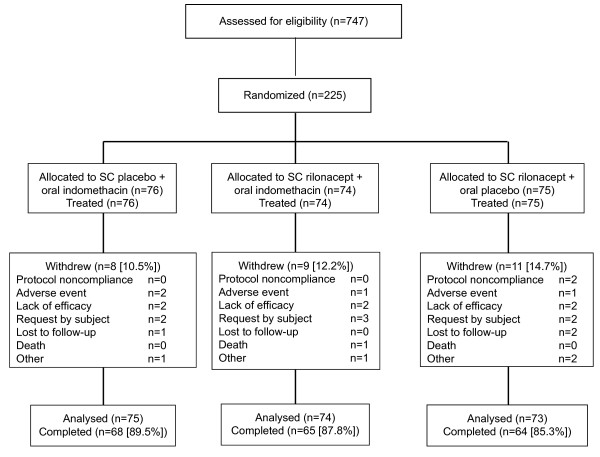
**Flow of patients through the study**. SC, subcutaneous.

Figure 1 shows the distribution and flow through the study of the 225 patients who were randomized to treatment. The demographic and clinical characteristics were generally similar among the treatment groups (Table 1) with a population that was predominantly male (94.1%) and white (75.7%), a mean (SD) age of 50.3 (10.6) years, and a body mass index of 33.1 (6.9), indicating obesity. Mean serum urate was 8.27 mg/dL, 15.8% of patients had visible tophi on examination, and 40.1% of patients had a history of ULT use. Prior use of analgesic medications (paracetamol or acetylsalicylic acid) as reported at baseline was low (8.2%).

**Table 1:** Baseline demographic and clinical characteristics

Variable	SC placebo + oral indomethacin (*n *= 75)	SC rilonacept + oral indomethacin (*n *= 74)	SC rilonacept + oral placebo (*n *= 73)
Age, years, mean (SD)	51.3 (10.9)	48.6 (10.0)	51.0 (10.8)
Gender, n (%)			
Male	71 (94.7)	71 (95.9)	67 (91.8)
Female	4 (5.3)	3 (4.1)	6 (8.2)
Race, n (%)			
White	54 (72.0)	54 (73.0)	60 (82.2)
Black or African American	15 (20.0)	15 (20.3)	11 (15.1)
Asian	5 (6.7)	2 (2.7)	2 (2.7)
Other	1 (1.3)	3 (4.1)	0
BMI, kg/m^2^, mean (SD)	32.1 (6.3)	33.5 (7.5)	33.7 (6.7)
Pain severity, mean (SD)			
Likert scale (0 to 4)	2.6 (0.7)	2.7 (0.7)	2.6 (0.7)
Numerical rating scale (0 to 10)	6.8 (2.2)	6.8 (2.0)	6.5 (2.3)
Duration of disease, years, mean (SD)	8.8 (6.7)	11.0 (7.9)	10.2 (9.9)
Prior number of gout flares per year, mean (SD)	4.8 (5.19)	5.5 (5.26)	5.2 (4.76)
Duration of a typical gout flare, days, mean (SD)	5.8 (3.6)	7.1 (4.2)	6.8 (7.3)
Tophi present, n (%)	10 (13.3)	12 (16.2)	13 (17.8)
Serum uric acid, mg/dL, mean (SD)	7.9 (1.9)	8.2 (2.1)	8.3 (1.7)
Prior medication use, n (%)			
Urate-lowering therapy	28 (37.3)	34 (45.9)	27 (37.0)
Analgesics (paracetamol or acetylsalicylic acid)	2 (2.7)	9 (12.2)	7 (9.6)

BMI, body mass index.

**Figure 2:**
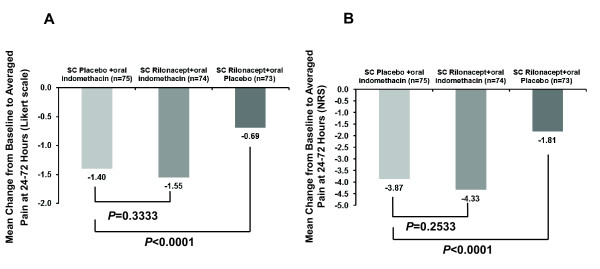
**Change in pain from baseline**. Data are mean change in pain of the index joint from baseline to pain averaged for the 24-, 48-, and 72-hour assessments for (**A**) the primary endpoint using a Likert scale (0 = no pain to 4 = extreme pain), and (**B**) using a numerical rating scale (NRS; 0 = no pain to 10 = extreme pain). SC, subcutaneous.

All treatment groups were observed to have significant reductions in pain from baseline when averaged at 24, 48 and 72 hours (*P *< 0.0001) and assessed using the Likert scale. However, the mean reduction in pain with rilonacept plus indomethacin, 1.55 points, was not statistically significantly greater than the mean reduction in pain with indomethacin alone, which was 1.40 points (least squares mean difference -0.14, 95% CI -0.44, 0.15, *P *= 0.333) (Figure 2A). Since the difference was not significant, formal comparison between pain reduction in the rilonacept monotherapy group, which was 0.69 points, and the indomethacin monotherapy group, which was 1.40 points, was not required. However, a separate ad hoc analysis for this comparison showed that the difference between indomethacin monotherapy and rilonacept monotherapy significantly favored indomethacin (*P *< 0.0001). Similar results were observed with the NRS; significant reductions from baseline were observed in each of the treatment groups (*P *< 0.0001), with pain reductions of 3.87 in the rilonacept plus indomethacin group, 4.33 in the indomethacin monotherapy group, and 1.81 in the rilonacept monotherapy group (Figure 2B). While the NRS change with rilonacept plus indomethacin was similar to that of indomethacin alone (*P *= 0.2533), the ad hoc analysis significantly favored indomethacin monotherapy relative to rilonacept monotherapy (*P *< 0.0001).

**Figure 3:**
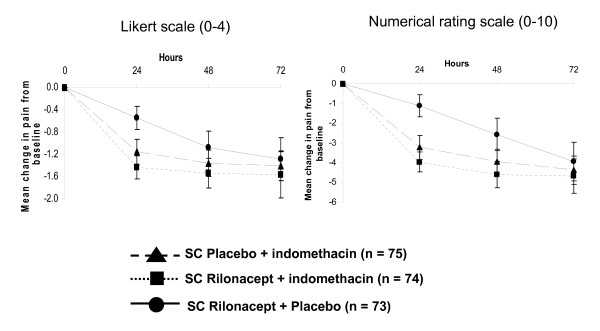
**Mean change in pain of the index joint from baseline at 24, 48, and 72 hours**. (**A**) Likert scale (0 = no pain to 4 = extreme pain). (**B**) Numerical rating scale (0 = no pain to 10 = extreme pain). SC, subcutaneous.

For the secondary endpoints of mean change in pain at 24, 48, and 72 hours (Figure 3), the trends in pain reduction among the three treatment groups were similar when pain was assessed using the Likert scale and the NRS; no significant differences were observed with rilonacept plus indomethacin relative to indomethacin monotherapy, but indomethacin monotherapy was significantly superior to rilonacept monotherapy at all time points (*P *< 0.05).

The proportion of patients who reported having taken rescue medication at > 24 to 48 hours in the rilonacept plus indomethacin group (3.0%) was similar to the indomethacin monotherapy group (4.3%).

**Figure 4:**
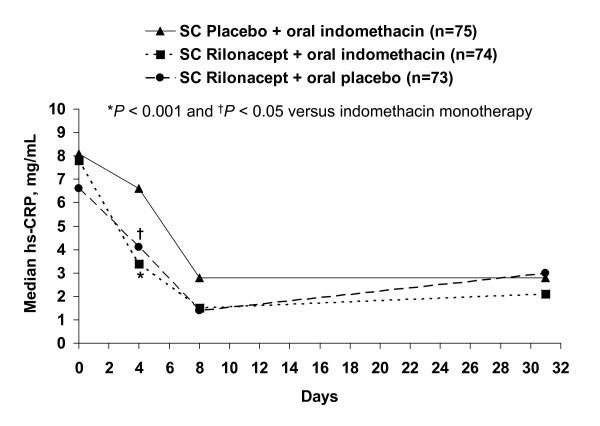
**Serum concentrations of high sensitivity C-reactive protein (hs-CRP) from baseline to day 31 (safety follow up)**. SC, subcutaneous.

All treatment groups were characterized by a general reduction from baseline in hs-CRP from initiation of treatment to the safety follow up at day 31 (Figure 4). At day 4, these reductions were significantly greater with rilonacept plus indomethacin (*P *< 0.0001) and rilonacept monotherapy (*P *= 0.0142) relative to indomethacin monotherapy.

Overall, the incidence of AEs was higher in the groups treated with rilonacept relative to indomethacin alone (Table 2); AEs were generally of mild to moderate severity. There were three patients with five serious AEs reported (hypertensive cardiomyopathy, myocardial infarction, ulcerative colitis, tubulointerstitial nephritis, and pyoderma gangrenosum), all in the rilonacept plus indomethacin group, which were not considered by the investigator to be related to treatment with rilonacept. One death was due to hypertensive cardiomyopathy that was not considered by the investigator to be related to treatment with rilonacept. Similar proportions of patients in each treatment group withdrew due to AEs, and the most frequently reported AEs were headache and dizziness, each of which occurred with a similar incidence across the treatment groups (Table 2).

**Table 2:** Treatment-emergent adverse events through safety follow up (day 31): incidence ≥ 5% in any treatment group

	Number (%) of patients		
	
Adverse event (AE)	SC placebo + oral indomethacin (*n *= 77)^1^	SC rilonacept + oral indomethacin (*n *= 73)^1^	SC rilonacept + oral placebo (*n *= 75)
Any AE	23 (29.9)	34 (46.6)	27 (36.0)
Discontinuations due to AE	2 (2.6)	1 (1.4)	1(1.3)
Serious AE	0	3 (4.1)	0
Headache	6 (7.8)	4 (5.5)	7 (9.3)
Dizziness	4 (5.2)	3 (4.1)	2 (2.7)

^1^Numbers differ from patient disposition since one patient was administered the wrong drug.

## Discussion

Whereas rilonacept has previously demonstrated efficacy in prevention of gout flare during initiation of ULT with allopurinol [34,35], the current study showed that adding rilonacept to indomethacin for the treatment of acute gout flares did not result in significantly greater pain relief compared to indomethacin alone over the 72-hour period following initiation of treatment. Importantly, the similarity of the magnitude of the pain reduction observed with indomethacin in this study to that reported in previous studies [38,39] buttresses the current data. Although rilonacept monotherapy was not formally compared with the other treatment groups, the reduction in pain at 24 and 48 hours with this regimen was clearly less than that achieved with the other treatment regimens, and was demonstrated to be significantly inferior to indomethacin monotherapy in the ad hoc analysis. However, improvement in pain at 72 hours for the three groups was similar. This may reflect a delayed effect of rilonacept or could simply reflect the self-limiting natural course of an acute gout flare [40].

Several factors may account for the observed results. In this study, rilonacept was administered within 48 hours of onset of an acute gout flare in patients who were already experiencing substantial pain. First, it is possible that rilonacept may have been more effective had it been administered earlier relative to flare onset, since the ability of IL-1 inhibition to effect a reduction in pain by blocking the cascade of downstream inflammatory mediators may depend on the timing of treatment. Results of a clinical trial with colchicine indicated the success of treating acute gout flares within 12 hours of onset [12]. Secondly, after a single SC injection, T_max _for rilonacept is approximately 48 to 72 hours. Since the primary endpoint was the change from baseline to the average of the pain assessments at 24, 48 and 72 hours, it is possible that drug concentrations at the gouty joint were insufficient during the majority of the primary endpoint assessment period. Furthermore, the size of rilonacept (approximately 250 kilodaltons) may limit the rate of distribution from plasma to target tissues involved in a gout attack. It is possible that, had rilonacept been administered by the intravenous route, higher serum concentrations achieved soon after administration could have driven greater drug penetration to target tissues, and hence, greater efficacy in treating acute gout flares.

These data appear to contrast with recent clinical trials of another IL-1 inhibitor, canakinumab, which demonstrated that IL-1 inhibition ameliorated inflammation and pain during an ongoing gout flare as compared to low-dose steroid injections [30,31]. Several reasons should be noted that may account for this difference, including that the canakinumab studies had several clinically relevant differences in study design and study populations. For example, the canakinumab studies were conducted in patients poorly responsive to, or unable to tolerate, NSAID and/or colchicine therapy. The duration of acute gout flare before therapy was allowed to be up to 5 days in each of the canakinumab studies [30,31] compared with only 48 hours in the current study of rilonacept. Prior to enrollment, patients in the current study were also specifically required to have tolerated NSAIDs and to have demonstrated symptomatic relief of gout flare pain with NSAIDs. The active comparator in the canakinumab studies was a corticosteroid, triamcinolone acetonide, administered as a single IM dose of 40 mg. This dose is lower than the single 60 mg IM dose previously suggested as being effective in two small clinical studies in acute gout [41,42], and although it was associated with partial pain relief in acute gout [30,31], the significance of the clinical effect is uncertain in the absence of comparison with placebo, or standard of care (colchicine or NSAIDs). It should also be noted that the indomethacin comparator in the current study has both potent analgesic as well as anti-inflammatory properties.

Given the collective distinctions in study designs, the extent of the intrinsic differences that may have contributed to the differing outcomes between the soluble decoy receptor rilonacept and the monoclonal antibody canakinumab are unclear. Differences in efficacy among agents in another class of anti-cytokine therapy, TNF-α antagonists, have been demonstrated to be related to such factors in arthritic diseases other than gout [43,44]. It remains to be addressed whether doses of rilonacept higher than employed in this study, selection of a different patient population, or a different comparator or route of administration (intravenous or intra-articular), might be more effective for treatment of acute gout flares. Interestingly, despite the lack of incremental efficacy with rilonacept for pain reduction compared with indomethacin in an acute gout flare, it should be noted that rilonacept, both as monotherapy as well as in combination with indomethacin, did result in significantly greater early reductions in hs-CRP than indomethacin monotherapy (*P *< 0.05). These reductions likely reflect IL-1 inhibition by rilonacept, and are consistent with those reported for canakinumab in acute gout flares [30].

Treatment with rilonacept in this clinical setting was generally well-tolerated, with the frequency of withdrawals due to AEs similar across treatment groups. Although the combination therapy group had a higher incidence of serious AEs relative to both of the monotherapy groups, these AEs were not considered related to rilonacept.

## Conclusions

In conclusion, in contrast with previous studies that have demonstrated efficacy of rilonacept for prevention of acute gout flares in patients initiating ULT, addition of rilonacept to an indomethacin treatment regimen and use of rilonacept alone provided neither significant additional pain relief nor superior pain relief, compared with indomethacin alone over the 72-hour period after treatment initiation in acute gouty arthritis.

## Abbreviations

AE: adverse event; hs-CRP: high sensitivity C-reactive protein; IL-1: interleukin-1; IM: intramuscular; MSU: monosodium urate; NSAID: non-steroidal anti-inflammatory drug; SC: subcutaneous; TNF: tumor necrosis factor; ULT: urate-lowering therapy.

## Competing interests

RT has consulted for Regeneron, Takeda, Savient, Novartis, Prescription Solutions, Pfizer, Ardea, BioCryst, and Metabolex. HRS has consulted for Regeneron, Novartis, Ardea, Pfizer, Metabolex, and Westward, and has received a grant from Takeda. JC has consulted for Regeneron. HB has been an investigator for Regeneron, Ardea, Novartis, Takeda, Metabolex, and Savient, has consulted for Regeneron, Ardea, Takeda, and Savient, and has spoken for Takeda, and Savient. RE, JW, SK, and SW are employees and stock holders of Regeneron Pharmaceuticals, Inc. Regeneron holds patents related to the content of the manuscript.

## Authors' contributions

RT, HRS, RE, JW, SK, SW made substantial contributions to the conception and design of the clinical trial. JC and HB were involved in the acquisition of data. All authors were involved in the analysis and/or interpretation of data. All authors were involved in drafting the manuscript and revising it critically for important intellectual content. All authors read and approved the final manuscript.
